# Does resveratrol affect prepared sperm parameters and chromatin quality in normozoospermic and asthenozoospermic patients before and after freezing? A lab trial study

**DOI:** 10.18502/ijrm.v13i9.7670

**Published:** 2020-09-20

**Authors:** Masoomeh Mohammadzadeh, Mohammad Ali khalili, Vahid Ramezani, Hamed Hamishehkar, Laleh Dehghan Marvast, Esmat Mangoli, Mahya Rajabi, Zhima Akhavan Sales, Ali Reza Talebi

**Affiliations:** ^1^Department of Reproductive Biology, Research and Clinical Center for Infertility, Yazd Reproductive Sciences Institute, Shahid Sadoughi University of Medical Sciences, Yazd, Iran.; ^2^Department of Pharmaceutics, Faculty of Pharmacy, Shahid Sadoughi University of Medical Sciences, Yazd, Iran.; ^3^Drug Applied Research Center, Tabriz University of Medical Sciences, Tabriz, Iran.; ^4^Abortion Research Center, Yazd Reproductive Sciences Institute, Shahid Sadoughi University of Medical Sciences, Yazd, Iran.; ^5^Department of Immunology, International Campus, Shahid Sadoughi University of Medical Sciences, Yazd, Iran.

**Keywords:** Resveratrol, Chromatin, Motility, Spermatozoa, Freeze

## Abstract

**Background:**

Previous studies have examined the effect of resveratrol as a potent antioxidant for free radicals in semen. While, the prepared spermatozoa are more affected by ROS factors due to centrifugation and incubation.

**Objective:**

To evaluate the RSV's effects on the prepared sperm parameters and chromatin quality in both normozoospermic and asthenozoospermic cases before and after freezing.

**Materials and Methods:**

The sample of 10 normozoospermic and asthenozoospermic men was prepared through the swim-up method. The groups were then divided into two samples of control and experimental (exposure to 30 µmol/l of RSV) to evaluate and compare the sperm parameters and chromatin quality before and after freezing.

**Results:**

The motility and viability of spermatozoa were seen to be significantly different before and after freezing separately in the control and treatment samples of the groups (p ≤ 0.001 and p = 0.001, respectively). However, the stated difference between the control and treatment samples of normozoospermic and asthenozoospermic patients were not significant (p > 0.05). In addition, the sperm morphology and chromatin quality were not significantly different between the two samples of each group; nonetheless, chromatin quality of the treated sample was better than that of the control before and after freezing.

**Conclusion:**

Despite the protective effects of RSV on the semen samples, RSV cannot affect significantly the prepared sperms parameters and chromatin quality in normozoospermic and asthenozoospermic patients.

## 1. Introduction

The high production rate of reactive oxygen species (ROS) is known as one of the main causes of male infertility, typically occurring when the ROS level is not in balance with that of the antioxidant physiology of semen, leading to oxidative stress (1, 2). Various factors may cause ROS, such as inclusive leukocytes, immature sperms, and abnormal sperm morphology. Furthermore, the environmental factors such as high oxygen pressure, incubation time, sperm preparation, reproductive techniques of assisted reproductive technology (ART), and sperm freezing can also be effective in ROS (3, 4).

The most important factors in ROS production in ART are sperm preparation and sperm freezing. Sperm freezing is done to maintain fertility, however, due to adverse changes during freezing and thawing processes, it damages the sperm viability and function. The production of osmotic pressure and the formation of ice crystals lead to the loss of water, making the conditions suitable for oxidative stress, instability, and decreased fluidity of the sperm membrane. Another consequence of freeze-thaw is the destruction of disulfide bands present in protamine, reducing the chromatin condensation and thus damaging the important components of sperm DNA. In fact, the main cause of oxidative stress is the imbalance between ROS produced during freezing and the antioxidants present in sperm (5, 6).

Sperm freezing removes the rich source of antioxidants from the semen. However, previous studies have shown that the application of antioxidants in culture media could protect the sperm from ROS's destructive effects (7, 8). One of these potent antioxidants, resveratrol (RSV), with a structure analogous to the estradiol, is associated with energy metabolism and production. In particular, it enhances adenosine monophosphate-activated protein kinase (AMPK) and reduces the rate of ROS and DNA apoptosis by preserving the mitochondrial membrane potential followed by semen freezing. Consequently, according to the effect of RSV on AMPK and the presence of AMPK at the whole flagellum and the post-equatorial region of the head, RSV has a main role in sperm motility (9, 10). Therefore, the freezing process has detrimental effects on the sperm parameters and chromatin quality (11). Because the IVF and ICSI processes are done by the prepared sperms lacking antioxidants during preparation, unlike semen, sperms are more affected by ROS factors due to centrifugation and incubation, ending in their impaired function. Many articles have suggested that the function of RSV is dose-dependent (12). However, no specific dose of this substance has been reported in the culture of human sperm preparation. Therefore, the aim of this study was to determine the specific dose of RSV and its effect on the sperms' parameters and chromatin before and after freezing in normozoospermic and asthenozoospermic individuals.

## 2. Materials and Methods

In this experimental study, the semen samples of 10 normozoospermic and 10 asthenozoospermic men aged 20-40yr who referred to Research and Clinical Center for Infertility, Yazd, Iran in October 2018 were used. Inclusion criteria were high semen volume, asthenospermic individuals, normospermic individuals, sperm with good morphology, mobility percentages for normospermic individuals up to 40-50 and in asthenic individuals 20-30, and sperm count between 10 and 70 million, the exclusion criteria included leukospermia, varicocele, diabetes, orchitis, azoospermia, and low semen volume.

After 48 hr of sexual abstinence, the samples were obtained by masturbation. Semen analysis was performed according to the World Health Organization (WHO) guidelines (13). The samples were prepared using the swim-up method (14). The prepared spermatozoa were divided into control (without exposure) and experimental (exposure to 30 µmol/l of RSV) samples (15). First, each sample (control and experimental) of normozoospermic and asthenozoospermic was analyzed separately after preparation (0 hr), 1 hr of incubation in 37°C and after freezing. Next, the sperm parameters and the chromatin quality of each sample before and after freezing were compared to investigate the effect of RSV.

### Sperm motility assessment

Sperm motility was evaluated visually. Different sperm motility rates were achieved, thanks to different percentages of progressive, nonprogressive, and immotile spermatozoa. Based on the WHO guidelines, 200 spermatozoa were determined and the percentage of motility was calculated under the light microscope using ×400 magnification (7, 13).

### Sperm morphology assessment

**Figure 1:**
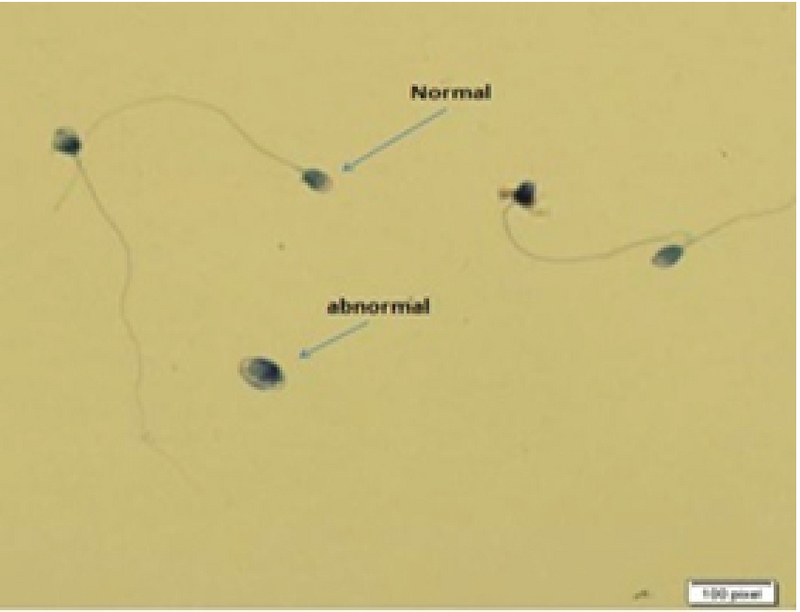
The morphology of the sperms detected by Diff-Quick staining (×1000 magnification).

Sperm morphology was evaluated by Diff-Quik staining based on the study of Kruger and colleagues and the WHO guidelines. Slides were prepared with 10 µl drop of sperm and a smear air-dried, stained with Diff-Quick. Later, 200 spermatozoa with normal and defected morphology were evaluated at a magnification of ×1000 (Figure 1) (16).

### Sperm viability assessment

**Figure 2:**
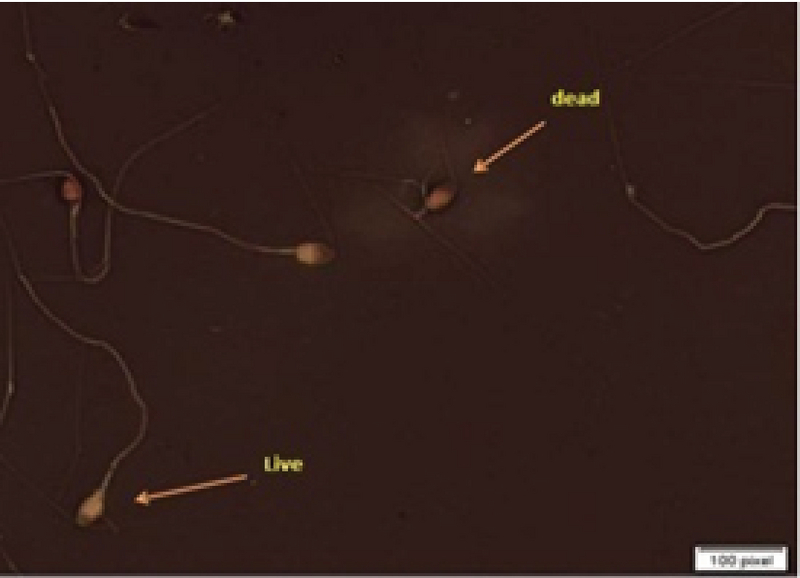
The viability of the sperms evaluated with eosin-nigrosine staining (×1000 magnification); live sperms are in white and dead sperms are in red.

Eosin-nigrosine staining was used for viability evaluation. The unstained sperms were assumed to be live; whereas, those with pink/red heads were considered dead. At least, 200 spermatozoa per patient were evaluated at a magnification of ×1000 (Figure 2) (17).

### Evaluation of sperm chromatin quality by Chromomysin A3 (CMA3)

**Figure 3:**
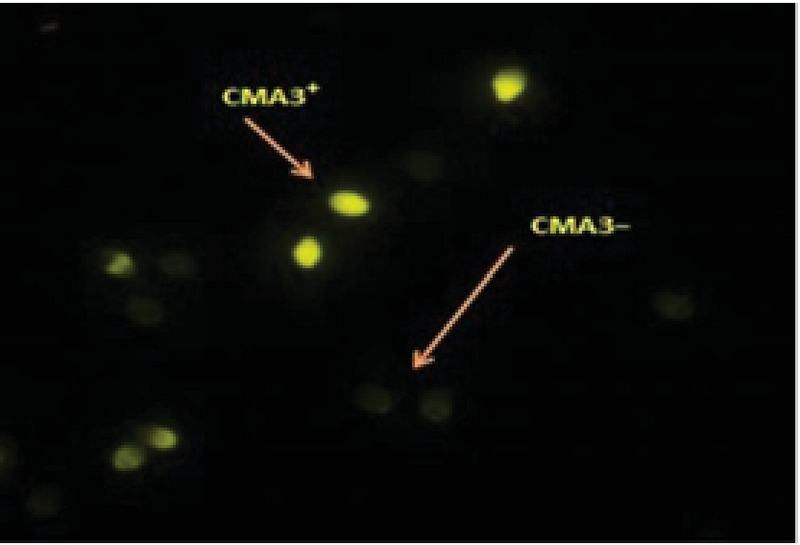
The protamine deficiency of sperm evaluated with CMA3 (fluorescent microscope, ×1000 magnification). Sperms with deficiency of protamine were stained yellow by CMA3 (CMA3+), while normal sperms were dark yellow.

Chromomysin A3 (CMA3) is a fluorescent dye, competing to guanosine cytosine-rich arrangements, and is used for the estimation of spermatozoa with protamine deficiency. Briefly, the thin smears of spermatozoa were prepared and fixed immediately with Carney's solution for 10 min at 4°C. Then, each slide was treated with 100 µL of CMA3 solution for 10 min in dark (Sigma-Aldrich, Germany). The slides were rinsed in McIrvin buffer and air dried. The analysis of the slides was performed using a fluorescent microscope (Olympus -Japan) with suitable filters (× 1000 magnification) (Figure 3) (18, 19).

### Cryopreservation and thawing of spermatozoa

Each sample was mixed with cryoprotectant in 1:1 ratio (Life Global, USA). The sperm samples were transferred into cryovials, cooled for 10 min at 4°C, transferred to liquid nitrogen vapors for 20 min, and then stored in liquid nitrogen. For warming the samples, they were transferred from liquid nitrogen to a water bath with ambient temperature and kept there until thawed. To remove cryoprotectant, the sperm samples were washed with hamsf10 medium (sigma, Germany) (1000 × gr, 10 min, room temperature) and suspended in hamsf10 (20). Finally, based on the aforementioned tests, the sperm parameters and chromatin quality were evaluated.

### Ethical Consideration

All participants signed a written consent form prior to the study. This experimental study was approved by the Ethics Committee of Shahid Sadoughi University of Medical Sciences (IR.SSU.MEDICINE.REC.1395.285).

### Statistical analysis

Considering the significant level of 5% and power test of 80% and the standard deviation of s = 24 for the RSV variable and the mean difference of 44, at least 10 people were needed. The data were analyzed by Graph pad prism. P < 0.05 was considered as the significant level. Moreover, the results of motility, morphology, and viability parameters were evaluated by ANOVA test, *t* test was used to evaluate the chromatin quality.

## 3. Results

**Figure 4:**
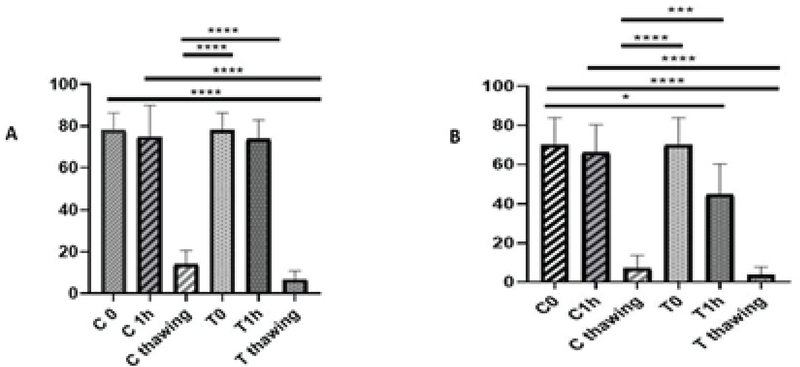
Comparison of motility of the samples after preparation, 1 hr of incubation at 37°C, and after freezing. (A) Normozoospermic group. (B) Asthenozoospermic group. C: Control; T: Treatment; ANOVA test.

The motility of the samples after preparation and 1 hr of incubation at 37°C was significantly different from that of them after freezing in each sample of normozoospermic and asthenozoospermic groups (p < 0.0001). The mean motility of each sample confirmed that the result before freezing was better than after. However, the difference between the mobility of each sample was not significant after preparation, 1 hr of incubation at 37°C, and after freezing in normozoospermic group (p = 0.99, p = 0.99, and p = 0.79, respectively) (Figure 4).

Interestingly, the results of viability were significantly different after preparation and 1 hr of incubation at 37°C than after freezing in the control and the treated samples of normozoospermic group (p = 0.000). However, these results were similar for asthenozoospermic group.

**Figure 5:**
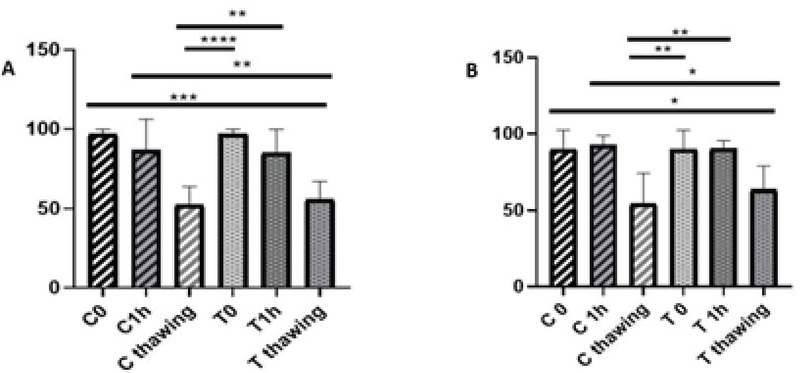
Comparison of the viability of the samples after preparation, 1 hr of incubation at 37°C, and after freezing. (A) Normozoospermic group. (B) Asthenozoospermic group. C: Control; T: Treatment; ANOVA test.

According to the viability test, the result after preparation and 1 hr of incubation at 37°C was significantly different than that after freezing in the control sample (p = 0.004 and p = 0.002, respectively). Furthermore, the results in the treatment sample of asthenozoospermic group were significantly different (p = 0.01 and p = 0.009, respectively). The comparison of each sample were not significantly different after preparation, 1 hr of incubation at 37°C and after freezing in normozoospermic and asthenozoospermic groups (p > 0.05) (Figure 5).

**Figure 6:**
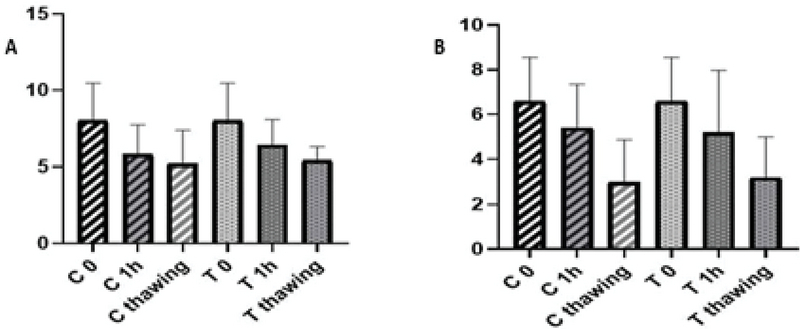
Comparison of morphology after preparation, 1 hr of incubation at 37°C, and after freezing. (A) Normozoospermic group. (B) Asthenozoospermic group. C: Control; T: Treatment; ANOVA test.

Further, the result of morphology showed no significant differences after preparation and 1 hr of incubation at 37°C than after freezing in the control and the treatment sample of normozoospermic group (p > 0.05). The result of morphology showed no significant differences after 1 hr of incubation at 37°C than after freezing, except in the case of preparation freezing in the control sample of asthenozoospermic (p = 0.029). However, no significant differences were observed after the preparation and 1 hr of incubation at 37°C compared to after freezing in the treatment sample of asthenozoospermic group (Figure 6).

Likewise, for the chromatin quality, no significant difference was observed after preparation and 1 hr of incubation at 37°C than freezing in each sample of normozoospermic; however, the mean of treatment samples in both groups showed that the result obtained before freezing was better than after freezing (Table I).

**Table 1:** Comparison of chromatin quality between study groups


	**Normozoospermic**			**Asthenozoospermic**		
**Groups**	**Control **	**Treatment **	**P-value**	**Control **	**Treatment **	**P-value**
**C1hr vs T 1hr**	75.6 ± 20.04	80.00 ± 13.73	0.98	44.40 ± 10.28	50.00 ± 10.28	0.94
**C thawing vs T thawing**	60.60 ± 13.73	74.20 ± 13.73	0.75	39.60 ± 10.28	52.20 ± 10.28	0.62
Data presented as Mean ± SE.* t* test, C: Control; T: Treatment						

## 4. Discussion

RSV is associated with energy metabolism by enhancing AMPK, which in turn preserves the mitochondrial membrane potential. As a result, it reduces the rates of ROS and DNA apoptosis followed by semen freezing. In this study, the concentration of 30 µmol/l RSV was selected as the most effective concentration. Despite the fact that RSV is known as a strong antioxidant in the literature, no significant difference was observed in the sperm parameters and chromatin quality between the control and treatment samples of each group. Various discussions can be run to explain this result. First, RSV might need other additional supplements for the semen contents to be effective. Therefore, RSV alone could not protect the prepared sperm against ROS. Furthermore, to enhance the RSV fusion to the sperm membrane, perhaps an appropriate carrier is required. As the last reason, RSV might have a long-term effect on the cell.

Another finding of our study was that the effect of RSV on sperm parameters and chromatin quality was more profound before freezing than after freezing in both normozoospermic and asthenozoospermic groups, attributable to the fact that the freezing process increased ROS (21). Consequently, this result can explain why RSV needs additional supplements to balance ROS. The rate of ROS was also lower in pre- than post-freezing; therefore, it could be concluded that freezing can cause devastating effects on sperm parameters and chromatin in exposure to large amounts of ROS.

Although many studies have investigated the effect of RSV, as an antioxidant, on the sperm function, the results of our research were inconsistent with other studies (15, 22-24). These inconsistencies can be due to the types of studied animal species, applied medium, and methods used in ART; as with regard to antioxidants, most studies were carried out on semen and not the prepared sperm. In line with our study, Shabani Nashteai and colleagues showed that 25 µm of RSV improved the cryopreserved sperm functions. However, they reached a different optimal concentration after freezing, which can be attributed to the fact that they studied semen including a wide variety of antioxidants and RSV.

Furthermore, the strong antioxidants contained in semen could better neutralize the effects of oxidative stress (10). However, this is contrary toour study; since our experiments were conducted on isolated sperms with no antioxidants from the semen. Meamar and colleagues demonstrated that the addition of 10 mM of RSV to cryopreservation media was not associated with significant differences in the sperm DNA fragmentation (25). This might be due to the high dose of RSV in medium or interactions between the antioxidants and the materials contained in cryopreservation media, probably reducing the effects of antioxidants or acting as toxic-like materials for the sperm.

Cui and coworker reported the effect of RSV on spermatozoa function in obese males. According to their results, 30 µmol/l of RSV had a significant effect on sperm function. The amount of dose selected in their study was similar to ours, however, the effect of RSV on sperm parameters was different in obese males after a 30-min exposure, probably due to the protective effects of RSV against hormonal abnormalities. In obese males, the considerably incremented level of estradiol could act as an exogenous estrogen, suppressing the destructive effects of intrinsic estrogen. Hence, it has a significant effect on the sperm function (15, 26).

Garcez and co-workers showed that RSV could avoid freezing damage (27). The different results achieved by them might be due to the type of applied cryopreservation media, which may produce less ROS compared to common methods. As mentioned earlier, when RSV was added to semen containing several different antioxidants, its functions were greatly affected. Contrary to our study, Garcez and co-authors showed that RSV minimized sperm DNA damage derived by cryopreservation only in infertile men, but not among the fertile men (27). This is because they used slow freezing method, which might have less risk factors on sperm compared to the rapid sperm freezing. However, the novelty of our study was the use of sperm preparation without antioxidants for exposure to RSV. We believe that several other studies are required to identify the functional mechanisms of RSV; for instance, future studies may deal with the status of chromatin quality and sperm parameters 4 and 24 hr after incubation to determine the long-term effects of RSV. On the other hand, due to time and budget constraints, we could not use RSV in other sperm factors including gene expression and fertility and the embryo development rate in these samples.

## 5. Conclusion

Despite all the protective effects of RSV on the motility and chromatin quality of the semen samples, it could not significantly affect the prepared sperms parameters and chromatin quality in normozoospermic and asthenozoospermic individuals. However, the chromatin quality of sperm was better in the treatment than the control groups.

##  Conflict of Interest 

The authors report no conflict of interest.
